# p53/p21 Pathway Involved in Mediating Cellular Senescence of Bone Marrow-Derived Mesenchymal Stem Cells from Systemic Lupus Erythematosus Patients

**DOI:** 10.1155/2013/134243

**Published:** 2013-09-16

**Authors:** Zhifeng Gu, Jinxia Jiang, Wei Tan, Yunfei Xia, Haixia Cao, Yan Meng, Zhanyun Da, Hong Liu, Chun Cheng

**Affiliations:** ^1^Department of Rheumatology, Affiliated Hospital of Nantong University, Nantong 226001, China; ^2^Department of Hematology, Affiliated Hospital of Nantong University, Nantong 226001, China; ^3^Department of Immunology, Medical College, Nantong University, Nantong 226001, China

## Abstract

Our and other groups have found that bone marrow-derived mesenchymal stem cells (BM-MSCs) from systemic lupus erythematosus (SLE) patients exhibited senescent behavior and are involved in the pathogenesis of SLE. Numerous studies have shown that activation of the p53/p21 pathway inhibits the proliferation of BM-MSCs. The aim of this study was to determine whether p53/p21 pathway is involved in regulating the aging of BM-MSCs from SLE patients and the underlying mechanisms. We further confirmed that BM-MSCs from SLE patients showed characteristics of senescence. The expressions of p53 and p21 were significantly increased, whereas levels of Cyclin E, cyclin-dependent kinase-2, and phosphorylation of retinoblastoma protein were decreased in the BM-MSCs from SLE patients and knockdown of p21 expression reversed the senescent features of BM-MSCs from SLE patients. Our results demonstrated that p53/p21 pathway played an important role in the senescence process of BM-MSCs from SLE.

## 1. Introduction

Systemic lupus erythematosus (SLE) is a chronic autoimmune disease characterized by multiorgan involvement and a remarkable variability in clinical presentations [1]. Previous studies have found that allogeneic MSCs transplantation (MSCT) used successfully in SLE achieved good efficacy [2–7]. However, Carrion and coworkers reported that autologous MSCT had no effect on disease activity in two SLE patients [8]. There are several studies that revealed that BM-MSCs from SLE patients showed impaired capacities of proliferation [9–11]. We have also found that BM-MSCs from both untreated and treated SLE patients showed prominent features of senescence, characterized by impaired capacities of proliferation, increased SA-*β*-gal activity, and disordered cytoskeleton distribution [12]. These findings suggested that the senescence of BM-MSCs from SLE patients may be a contributing factor to disease pathogenesis.

It has been reported that cell cycle relation proteins such as p53/p21^Cip1^, p16^INK4A^/Rb, and Pten/p27^Kip1^ were involved in the cellular senescence process [12–14]. We previously observed that BM-MSCs from SLE patients showed increased expression of p16^INK4A^, knockdown of p16^INK4A^ expression increased proliferation capacities, and decreased SA-*β*-gal activity; it suggested that cell cycle relation protein p16^INK4A^ was involved in the cellular senescence process of BM-MSCs from SLE patients. While after knockdown of p16^INK4A^ expression, the senescence features of BM-MSCs from SLE patients were not fully reversed [12]. That implied that there were also other cell cycle relation proteins involved in regulating cell senescence of BM-MSCs from SLE patients. Recently, studies have shown that p53/p21 pathway played an important role in regulating the cell senescence progress of MSCs [15–17]. The discovery that upregulation of the p53 pathway may have a critical role in mediating the reduction proliferation of human MSCs was also reported [17]. These data suggested that p53/p21 pathway took a part in regulating cell senescence of BM-MSCs. However, whether p53/p21 pathway was closely associated with the senescence of BM-MSCs from SLE patients has not been explored.

In the present study, we further clarified that BM-MSCs from SLE patients showed prominent features of senescence. We also found that the expressions of p53 and p21 were significantly increased, while the levels of Cyclin E, cyclin-dependent kinase-2 (CDK2), and phosphorylation of retinoblastoma protein (p-Rb) expression were decreased in BM-MSCs from SLE patients. Furthermore, we found that the expressions of p53 and p21 were significantly increased in nucleus of BM-MSCs from SLE patients, while the expressions of Cyclin E and CDK2 were significantly decreased in nucleus of BM-MSCs from SLE patients. Knockdown of p21 expression could reverse the senescent behavior of BM-MSCs from SLE patients. In our current study, our data showed that the cell senescent of BM-MSCs in SLE patients may get through the accumulation of p53 and p21 proteins.

## 2. Materials and Methods

### 2.1. Patients

Twenty-two female SLE patients aged 14–42 years (mean 27.73 ± 8.81 years) were enrolled in the study and retrieved from the archival files of the Department of Rheumatology, Affiliated Hospital of Nantong University from 2010 to 2012. The clinical features of patients summarily are shown in Table 1. The SLE diagnosis was made based on the criteria proposed by the American College of Rheumatology. The Systemic Lupus Erythematosus Disease Activity Index (SLEDAI) was used to measure disease activity [18]. Using a cutoff SLEDAI score of 8; all patients were categorized as active. Twenty-two healthy subjects were included as normal controls. All patients were females, and their age distribution was similar to that of the cases. All patients and controls gave consent to the study, which was approved by the Ethics Committee of the Affiliated Hospital of Nantong University.

### 2.2. Isolation of BM-MSCs from Bone Marrow and Cell Culture

BM-MSCs were isolated and cultured as we have reported previously [12]. Five milliliters of BM was mixed with an equal volume of phosphate-buffered saline (PBS). Then, the resuspended cells were layered over Ficoll solution (1.077 g/mL) and centrifuged at 2,000 rpm for 20 minutes at room temperature. The mononuclear cells were collected at the interface. Next, the cells were resuspended in low-glucose Dulbecco Modified Eagle Medium (L-DMEM) supplemented with 10% heat inactivated fetal bovine serum (FBS). The cell viability was determined by trypan blue exclusion. Then, the cells were counted and plated at a density of 2 × 10^7^ cells per 25 cm^2^ dish. The cultures were maintained at 37°C in a 5% CO_2_ incubator, and the medium was changed after 48 hours and every three days thereafter. When the BM-MSCs were confluent, the cells were recovered by the addition of 0.25% trypsin-EDTA. The cells were then replanted at a density of 1 × 10^6^ cells per 25 cm^2^ dish. Flow cytometric analysis showed that the cells were positive for CD29, CD44, CD105, and CD166 but negative for CD14, CD34, CD38, CD45, and HLA-DR [12]. After 3 passages (p3), cells were used for the following studies.

### 2.3. Proliferation Assays

Cell proliferation was measured using the commercial Cell Counting Kit (CCK)-8 assays in accordance with the manufacturer's instructions. Briefly, cells were seeded onto 96-well cell culture cluster plates (Corning Inc., Corning, NY, USA) at a concentration of 2 × 10^4^ cells/well in volumes of 100 *μ*L, and grown overnight. Cell Counting Kit-8 reagents (Dojindo, Kumamoto, Japan) were added to a subset of wells under different treatment and incubated for 2 h at 37°C, and absorbance was quantified using an automated plate reader.

### 2.4. Assay for Colony Forming Unit (CFU)

BM-MSCs were plated at densities of 1000, 500, 250, 100, 50, and 25 cells/cm^2^ in 24-well dishes. Cells were cultured for fifteen (15) days before they were fixed and stained with 1% crystal violet in methanol. Colonies with diameters larger than 3 mm were considered for counting.

### 2.5. Cell Cycle Analyses

For cell cycle analysis, cells were fixed in 70% ethanol for 1 h at 4°C and then incubated with 1 mg/mL RNase A for 30 min at 37°C. Subsequently, cells were stained with propidium iodide (50 mg/mL; Becton Dickinson, San Jose, CA, USA) in phosphate-buffered saline (PBS), 0.5% Tween-20, and analyzed using a Becton Dickinson flow cytometer BD FACScan (San Jose, CA, USA) and Cell Quest acquisition and analysis programs. Gating was set to exclude cell debris, cell doublets, and cell clumps.

### 2.6. SA-*β*-gal Assay

The SA-*β*-gal assay was used to detect cell senescence. The SA-*β*-gal activity was determined using a kit from the Chemical Company following the manufacturer's instructions. In brief, cells were cultured on slips in the 24-well plates overnight and fixed with paraformaldehyde. After incubated with SA-*β*-gal overnight, the slips were washed and analyzed under the microscope.

### 2.7. Western Blotting

To assay p53, p21, Cyclin E, CDK2, Rb, and p-Rb protein, the total cellular proteins was extracted through the following methods: BM-MSCs were washed in cold-buffered PBS and were then lysed in RIPA buffer (150 mM NaCl, 1% Triton X-100, 0.5% NaDOD, 0.1% SDS, and 50 mM Tris, pH 8.0). After centrifugation (12,000 rpm, 5 min) at 4°C, the protein supernate was transferred into new tubes. The protein concentration of the samples was determined with a bicinchoninic acid protein assay (Pierce, USA). Equal amounts of protein were resolved using 10% SDS-PAGE and transferred onto polyvinylidene difluoride (PVDF, Millipore, USA) membranes. The membranes were blocked with 5% dried skim milk in TBST (20 mM Tris, 150 mM NaCl, 0.05% Tween-20). After 2 h at room temperature, the membranes were incubated overnight with polyclonal antibody. Antibodies used were as follows: anti-p53 (1 : 500; Santa Cruz Biotechnology); anti-p21 (1 : 500; Santa Cruz Biotechnology); anti-Cyclin E (1 : 500; Santa Cruz Biotechnology); anti-CDK2 (1 : 1000; Santa Cruz Biotechnology); anti-Rb (1 : 500; Santa Cruz Biotechnology); and anti-p-Rb (1 : 500; Santa Cruz Biotechnology). Then, horseradish peroxidase-linked IgG was used as the secondary antibody. Immunoreactive bands were visualized by chemiluminescence (NEN Life Science Products, Boston, MA, USA). After the chemiluminescence was exposed to X-ray films, the films were scanned using a Molecular Dynamics densitometer (Imaging Technology, ON, Canada). The experiments were carried out on three separate occasions.

### 2.8. Immunofluorescence

Immunofluorescence was used to examine the lactation and expression of p53, p21, Cyclin E and CDK2 in BM-MSCs. At p3, the cells were seeded onto 25 mm dishes and cultured for 24 h. After washing with PBS, BM-MSCs were fixed with 4% paraformaldehyde (PFA) and the cells were blocked in 1% bovine serum albumin (Sigma-Aldrich, St. Louis) and 0.2% Triton-100 (Sigma-Aldrich) and then incubated at 37°C for 1 h with primary antibody to p53 (anti-rabbit, 1 : 100, Santa Cruz), p21 (anti-mouse, 1 : 200, Santa Cruz), CDK2 (anti-rabbit, 1 : 200, Santa Cruz), and Cyclin E (anti-mouse, 1 : 200, Santa Cruz). Then, the cells were washed and incubated in the dark for 1 h at 37°C with goat anti-rabbit- (cy3-) conjugated antibodies (1 : 300, ICN Cappel, USA) or goat anti-mouse FITC-conjugated antibodies (1 : 300, Dako, USA); the nuclei were counterstained with DAPI. After being washed and mounted, the cells were examined under a fluorescence microscope.

### 2.9. Separation of the Nuclei and Cytoplasm

To assay the p53, p21, Cyclin E, and CDK2 proteins, cytoplasmic and nuclear proteins from cultured cells were prepared using NE-PER nuclear and cytoplasmic extraction reagents (Pierce Chemical Company, USA), respectively. *β*-actin and *β*-tubulin were used as the internal control for the cytoplasmic and nuclear proteins. Cells were lysed in ice-cold hypotonic buffer (10 mM HEPES, 1.5 mM MgCl_2_, 10 mM KCl, 0.5 mM DTT, 0.5 mM phenylmethylsulfonyl fluoride, and 0.625% Nonidet P-40) for 15 minutes on ice. After vortexing for 10 seconds, the lysate was centrifuged for 10 minutes at maximum speed to obtain the cytoplasmic fraction in the supernatant. The remaining pellet was incubated with hypertonic buffer (20 mM HEPES, 420 mM NaCl, 25% glycerol, 0.5 mM DTT, 0.5 mM phenylmethylsulfonyl fluoride, and 0.12 mM Aprotinin per mL) for 60 minutes on ice and then centrifuged to obtain the supernatant containing the nuclear fraction. The protein concentration of the samples was determined by a bicinchoninic acid protein assay (Pierce, USA). The cytoplasmic fraction, the nuclear fraction, and the whole-cell lysates were used for western blot analysis as described previously. Antibodies used were as follows: anti-*β*-actin (1 : 600; Santa Cruz Biotechnology); anti-*β*-tubulin (1 : 600; Santa Cruz Biotechnology); and p53, p21, Cyclin E, and CDK2 protein antibodies as described previously.

### 2.10. siRNAs and Transfection

A double-stranded RNA that targeted human p21 and a nonsilencing control siRNA were obtained from Santa Cruz Biotechnology. The transfection of the BM-MSCs with the synthetic siRNA was performed using the Lipofectamine 2000 reagent (Invitrogen) according to the manufacturer's instructions. The cells were assayed after 48 h of transfection. For the mock transfection, the procedure was performed in the absence of the siRNA duplex.

### 2.11. RNA Preparation and RT-PCR

Total RNA of BM-MSCs cells were extracted using a Trizol extraction kit according to the manufacturer's procedure. Total RNA was reverse-transcribed using the Thermo Script RT-PCR system (Invitrogen). Primers pairs for p21 were sense, 5′-CAGAATCACAAACCCCTA-3′, and antisense, 5′-TGTTTTGAGTAGAAGAAT-3′. Cycling conditions were 94°C for 45 s, 55°C for 45 s, 72°C for 30 s, and a total of 30 cycles. Glyceraldehyde-3-phosphate dehydrogenase (GAPDH) was used as internal control and was detected using the primers sense, 5′-TGATGACATCAAGAAGGTGGTGAAG-3′, and antisense, 5′-TCCTTGGAGGCCATGTGGGCCAT-3′. Cycling conditions were 94°C for 30 s, 55°C for 30 s, 72°C for 30 s, and a total of 28 cycles. The PCR products were electrophoresed through a 1.5% agarose gel and visualized with ethidium bromide staining. The relative differences in the expression levels were normalized using GAPDH.

### 2.12. Statistical Analysis

The density of bands in Western blots or RT-PCR were measured with image analysis system. All of the results were representative of three independent experiments. All data were presented as mean ± standard deviation (SD) of the replicates and were analyzed by Student's *t*-test with *P* values less than 0.05 considered statistically significant. All of the statistical analyses were performed using SPSS 11.0 software.

## 3. Results

### 3.1. BM-MSCs from SLE Patients Showing Prominent Feature of Senescence

**Figure 1:**
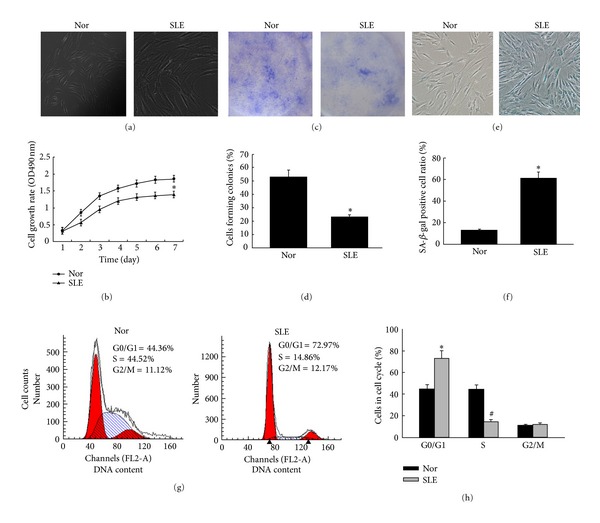
BM-MSCs from SLE patients are aging cells. (a) At p3, the morphology of normal BM-MSCs showed homogeneous spindle-shaped fibroblast-like growth. However, BM-MSCs from SLE patients appeared bigger in size and flattened in appearance. (b) Growth curve of BM-MSCs was tested by cell counting assay. The absorbance was shown as the proliferation rate. BM-MSCs from SLE patients grew more slowly than those from the control group. Each point represents quantities relative to the normal group at Day 1 (*P < 0.05). ((c)-(d)) Counted colonies of BM-MSCs from SLE patients and normal control were plated at a density of 25 cells = cm^2^ for 15 days in culture, stained with 1% crystal violet. Each bar represents quantities relative to BM-MSCs from Nor group and is mean ± SD of three experiments (*P < 0.05). ((e)-(f)) SA-*β*-gal was used to examine BM-MSCs senescence. BM-MSCs from SLE patients and normal control were performed SA-*β*-Gal staining. The number of SA-*β*-gal-positive cells obviously increased in BM-MSCs from SLE patients compared with those of normal control. Each bar represents quantities relative to BM-MSCs from Nor group and is mean ± SD of three experiments (*P < 0.05). (g) After cells culture, cells were removed from the culture dish with trypsin/EDTA at p3, fixed, stained for DNA with PI, and analyzed by flow cytometry (*y*-axis, cell count; *x*-axis, DNA content). (h) Mitotic indices of BM-MSCs from control group and SLE patients. Graphs in (h) represent DNA content indicating the percentages of cells in G0/G1, S, and G2/M phases of the cell cycle. The data are obtained from staining the DNA of ASCs from normal group and SLE patients. A decrease in the percentage of cells in S phases was seen in SLE patients, while an increase in percentage of cells in G0/G1 phase was seen in SLE patients. Each bar represents quantities relative to BM-MSCs from Nor group and is mean ± SD of three experiments (*P < 0.05) (^#^ *P* < 0.05). Following the cell cycle progression, these cells showed a greater fraction in quiescent of G0/G1 phase in SLE patients when compared with normal control.

As we have studied previously, BM-MSCs from SLE patients appeared bigger in size and flattened in appearance (Figure 1(a)). From growth curve, we found that BM-MSCs from SLE patients grew more slowly than those from the normal group (Figure 1(b), *P* < 0.05). Simultaneously, colony-forming unit (CFU) potential of BM-MSCs declined by about a quarter in SLE patients compared to normal group (Figures 1(c)-1(d), *P* < 0.05) indicating that the capability of replicating and forming colonies of BM-MSCs from SLE patients were decreased. Furthermore, we have found that the number of SA-*β*-gal-positive cells was notably increased in BM-MSCs from SLE patients, which was used to examine MSCs senescence. The cell count revealed that the numbers of SA-*β*-gal-positive cells from SLE patients were obviously higher than those of normal group (Figures 1(e)-1(f), *P* < 0.05). Beyond these, the cell cycle distribution of BM-MSCs was determined by FACS analysis following propidium iodide staining of cellular DNA showed that there were more BM-MSCs restricted in the G1 phase which were harvested from SLE patients than that of normal group (44.36 ± 2.36% versus 72.89 ± 3.21%, Figures 1(g)-1(h)). These data indicated that the BM-MSCs from SLE patients were senescent cells, which were similar to previous studies [12].

### 3.2. The p53/p21 Pathway Plays an Important Role in Cell Senescence of BM-MSCs from SLE Patients

**Figure 2:**
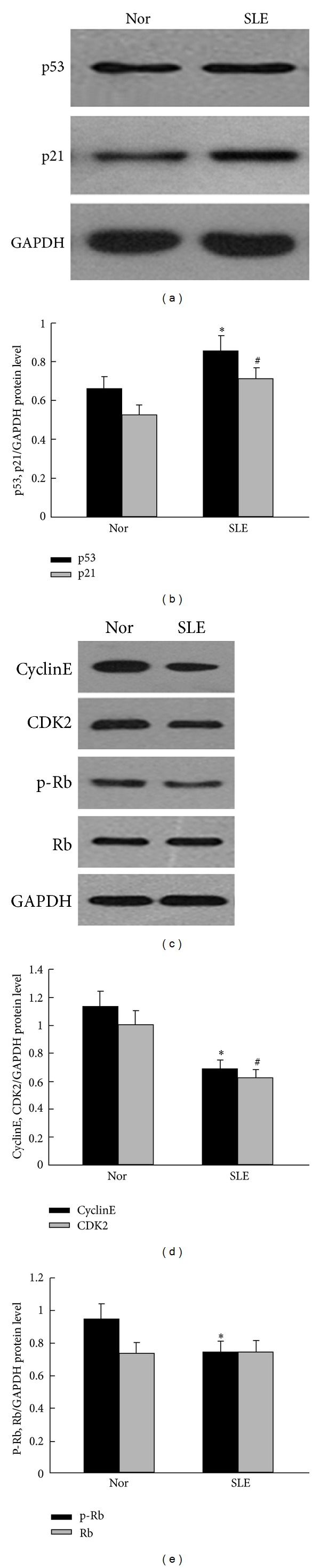
The expression of p53/p21 and cell cycle-related molecules in BM-MSCs from SLE patients. Western blot was used to analyze p53/p21 and their relative proteins expressions. (a) The expression of p53 and p21 was significantly increased in BM-MSCs from SLE patients. (b) Quantification of p53 and p21 protein levels: the relative levels of protein expressions were normalized to GAPDH expression. Values are means ± SD of three experiments (*P < 0.05; ^#^ *P* < 0.05). (c) Western blot analysis and the expressions of Cyclin E, CDK2, and p-Rb in BM-MSCs from SLE patients were decreased. (d) Quantification of Cyclin E and CDK2 protein levels: the relative levels of protein expressions were normalized to GAPDH expression. Values are means ± SD of three experiments (*P < 0.05; ^#^ *P* < 0.05). (e) Quantification of p-Rb and Rb protein levels: the relative levels of protein expressions were normalized to GAPDH expression. Values are means ± SD of three experiments (*P < 0.05). GAPDH was used as the internal control. Total p53 and p21 were significantly increased in BM-MSCs from SLE patients, while the expressions of Cyclin E, CDK2, and p-Rb were significantly decreased in BM-MSCs of SLE patients when compared with those in normal control.

It is reported that the p53/p21 pathway plays an important role in regulated BM-MSCs senescence process. In the present study, we found that the expressions of p53 and p21 were increased in the BM-MSCs from SLE patients (Figures 2(a)-2(b); *P* < 0.05, resp.), while the expressions of Cyclin E and CDK2 were markedly decreased in BM-MSCs from SLE patients (Figures 2(c)-2(d); *P* < 0.05, resp.). Moreover, a reduced phosphorylation of Rb in MSCs from SLE patients was detected (Figures 2(c) and 2(e); *P* < 0.05).

### 3.3. p53 and p21 Were Made Function That Depends on Mainly Localization in Nuclear Fraction of the BM-MSCs from SLE Patients

**Figure 3:**
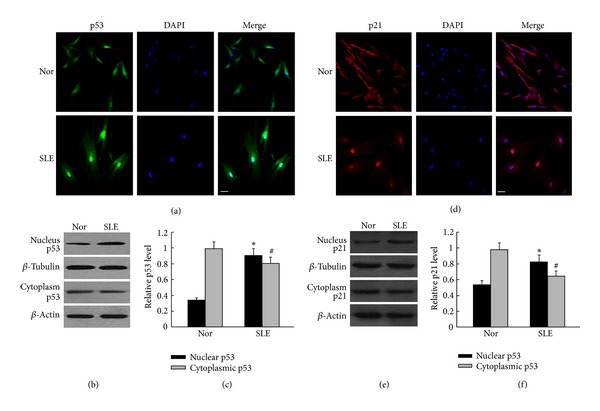
Analysis of the location and expression of p53 and p21 in the cytoplasmic and nuclear of BM-MSCs from SLE patients. ((a), (d)) Immunofluorescence staining to analyze the location of p53 and p21 in BM-MSCs. In the BM-MSCs from SLE patients, there was a clear increase in nuclears p53 and p21 expression. The scale bar is 25 mm. ((b), (e)) Western blot analysis of the cytoplasmic and nuclear p53 and p21 expressions. *β*-Actin was used as the internal control for the cytoplasmic proteins, whereas *β*-tubulin was used as the internal control for the nuclear proteins. ((c), (f)) Quantification the expressions of p53 and p21 in the nuclear and cytoplasmic. Compared with normal control, the location and expression of p53 and p21 in the nuclear of BM-MSCs were significantly higher in SLE patients. The relative levels of protein expressions were normalized to *β*-actin expression. Values are means ± SD of three experiments (*P < 0.05).

**Figure 4:**
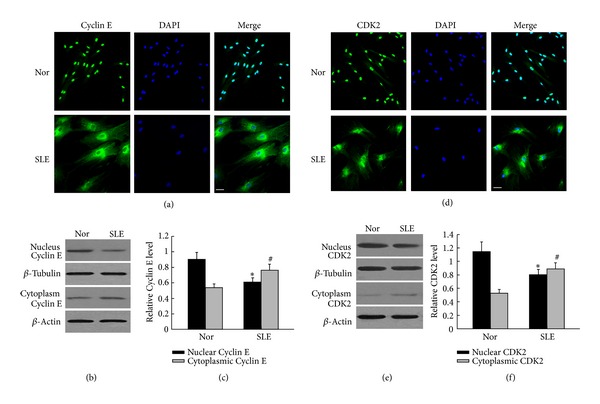
Analysis of the location and expression of Cyclin E and CDK2 in the cytoplasmic and nuclear of BM-MSCs from SLE patients. ((a), (d)) The locations of Cyclin E and CDK2 in BM-MSCs were tested by immunofluorescence staining. We found that the expression of Cyclin E and CDK2 was notably decreased in nuclear of BM-MSCs from SLE patients, while there were notably higher expressions in nuclears in normal control. The scale bar is 25 mm. ((b), (e)) Western blot to analyze the expressions of Cyclin E and CDK2 in cytoplasmic and nuclear. ((c), (f)) Quantification the expressions of Cyclin E and CDK2 in the nuclear and cytoplasmic. Compared with normal control BM-MSCs, Cyclin E and CDK2 in the nuclear were significantly lower in SLE patients; the relative levels of protein expressions were normalized to *β*-actin expression. Values are means ± SD of three experiments (*P < 0.05).

Recent studies have found that protein and its function were based on a subcellular localization. The p53 and p21 proteins which accumulated in the nucleus are necessary for cell cycle arrest. In our study, tested by immunofluorescence staining, we observed that p53 and p21 were mainly localized in the nuclei of the BM-MSCs from SLE patients, whereas lower levels were found in the cytoplasm of the BM-MSCs from SLE patients than that of the normal control (Figures 3(a) and 3(d)). To further detect the levels of these proteins' expression, we used separation of the nuclei and cytoplasm and Western blot analysis. We found that p53 and p21 were expressed more in the nuclei of the BM-MSCs from SLE patients, whereas lower levels were found in the cytoplasm of the BM-MSCs from SLE patients than that of the normal control (Figures 3(b), 3(c), 3(e), and 3(f); *P* < 0.05, resp.). In the meantime, we observed that Cyclin E and CDK2 were mainly localized in the cytoplasm and lower levels were found in the nuclei of BM-MSCs from SLE patients than that of the normal control (Figures 4(a) and 4(d); resp.). Furthermore, we found that, Cyclin E and CDK2 were expressed more in the cytoplasm of the BM-MSCs from SLE patients, whereas lower levels were found in the nuclei of the BM-MSCs from SLE patients than that of the normal control (Figures 4(b), 4(c), 4(e) and 4(f); *P* < 0.05, resp.). The results suggested that p53, p21, and CyclinE-CDK2 effect on BM-MSCs senescence process of SLE patients might base on them subcellular localization.

### 3.4. Knockdown of p21 Expression Reversed Feature Senescence of BM-MSCs from SLE Patients

**Figure 5:**
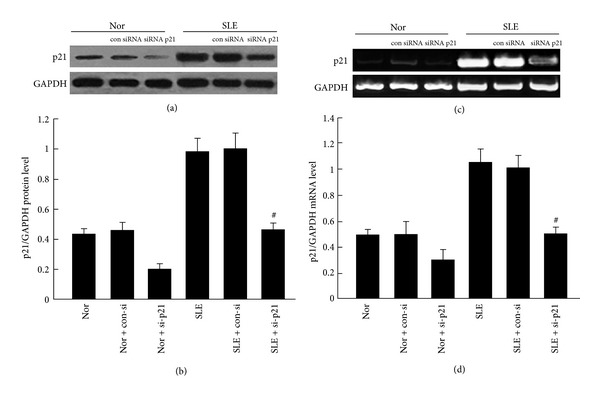
p21 siRNA decreased p21 expression in BM-MSCs. Cells were transfected with p21 siRNA for 24 hours. p21 expression was significantly decreased in BM-MSCs both in SLE patients and normal control by immunoblot analyses ((a)-(b)) and RT-PCR ((c)-(d)). The relative levels of protein expressions and gene expression of target mRNA were normalized to GAPDH expression. Values are means ± SD of three experiments (^#^ *P* < 0.05).

**Figure 6:**
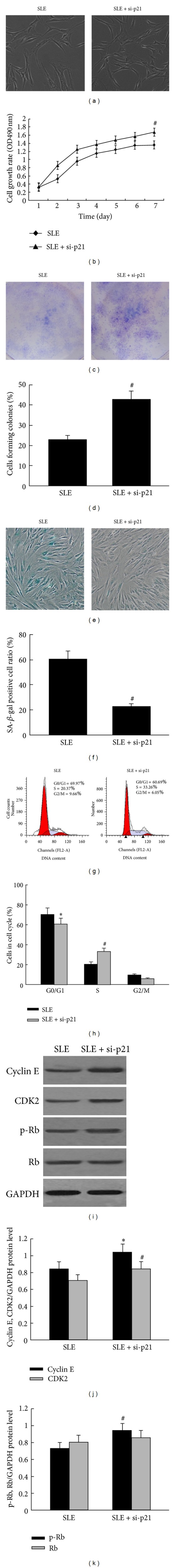
p21 knockdown reversed the ageing characteristics of BM-MSCs from SLE patients. (a) The morphology of BM-MSCs showed more spindle-shaped fibroblast-like growth after knockdown p21 when compared with BM-MSCs culture without si-p21. (b) Growth curve of BM-MSCs treated with and without si-p21 was tested by cell-counting assay. It has showed that, when p21 was knocked down, the cell proliferation rate was increased. Each point represents quantities relative to the BM-MSCs from SLE treated without si-p21 at Day 1 (*P < 0.05) (^#^ *P* < 0.05). ((c)-(d)) CFU of BM-MSCs from treated with si-p21 were increased; each bar represents quantities relative to BM-MSCs from SLE treated without si-p21 and is mean ± SD of three experiments (^#^ *P* < 0.05). ((e)-(f)) After treated with si-p21, the number of SA-*β*-gal-positive cells was obviously decreased; each bar represents quantities relative to BM-MSCs from SLE treated without si-p21 and is mean ± SD of three experiments (^#^ *P* < 0.05). (g) DNA content between BM-MSCs culture with and without si-p21 from SLE patients was compared by flow-cytometry. (h) When BM-MSCs from SLE patients treated with si-p21, the percentage of cells in G0/G1 was obviously decreased, and increase in percentage of cells in S phases was seen in SLE patients. Each bar represents quantities relative to BM-MSCs from SLE treated without si-p21 and is mean ± SD of three experiments (*P < 0.05; ^#^ *P* < 0.05). ((i)–(k)) Expressions of cyclin E, CDK2, and p-Rb in BM-MSCs from SLE patients were tested by Western blot analyses and quantification analyses between culture with and without si-p21. The relative levels of protein expressions were normalized to GAPDH expression. Values are means ± SD of three experiments (*P < 0.05; ^#^ *P* < 0.05).

To further assess the role of p53/p21 in the BM-MSCs senescence progress, we have transfected the BM-MSCs with p21 siRNA and a nonspecific siRNA. As predicted, the p21 expression was considerably decreased in the p21 siRNA-transfected BM-MSCs from SLE patients (Figure 5, *P* < 0.05). To investigate the role of p21 in BM-MSCs senescence, BM-MSCs were cultured with or without p21 siRNA. At p3, the morphology of BM-MSCs from SLE patients culture in p21 siRNA appeared a fibroblast-like morphology (Figure 6(a)); moreover, cell proliferation assay showed that the proliferation rate of p21 knockdown BM-MSCs from SLE patients was increased (Figure 6(b)) and CFU potential of BM-MSCs from SLE patients cultured with p21 siRNA increased by about a half (Figures 6(c)-6(d), *P* < 0.05). Meanwhile, compared to BM-MSCs from SLE patients cultured without p21 siRNA, SA-*β*-gal-positive cells were notably decreased and about a quarter of the cells were stained positive in p21-knockdown BM-MSCs from the SLE patients (Figures 6(e)-6(f), *P* < 0.05). Furthermore, for cell cycle distribution of BM-MSCs, there were more cells restricted in the S phase harvested from BM-MSCs after p21 knockdown (20.37 ± 3.25% versus 33.26 ± 3.54%, Figures 6(g)-6(h)). Meanwhile, we also detected that the expressions of Cyclin E, CDK2, and p-Rb were increased in BM-MSCs treatment with si-p21 (Figures 6(i)–6(k), *P* < 0.05). These results implied that the p53/p21 pathway plays an essential role in BM-MSCs ageing of SLE patients.

## 4. Discussion

In our study, we further confirmed that BM-MSCs from SLE patients showed prominent features of senescence, which were characterized by increased cell size, decreased proliferation and colony forming potential, more cells restricted in the G0/G1 phase, and increased SA-*β*-gal activity. We found that the expressions of p53 and p21 were significantly increased, while levels of Cyclin E, CDK2, and p-Rb expressions were decreased in BM-MSCs from SLE patients. We also found that the expressions of p53 and p21 were significantly increased, while Cyclin E and CDK2 were significantly decreased in nucleus of BM-MSCs from SLE patients. Knockdown p21 expression could reverse the senescent behavior of BM-MSCs from SLE patients.

Recently, research showed that p53/p21 pathway plays an important role in the cell senescent [19–23]. It has been reported that cell cycle progression is regulated by cyclins and CDK, such as Cyclin E and CDK2, and this regulation is negatively inhibited by tumor suppressors, such as p53 and CDK inhibitors p21, a target gene of p53 protein [24]. Elmore found that overexpression of p53 causes arrest of cell growth [25]. p21 is a key molecule in cell cycle regulation that binds to and inhibits the activity of CDK complexes, thereby that inhibits Rb phosphorylation. The phosphorylation of Rb is a well-described regulator of the cell cycle [26]. It has also been demonstrated that the p21 accumulates progressively in aging cells, binds to, and inactivates all Cyclin E-CDK2 complexes, which is responsible for the phosphorylation of the Rb results in irreversible G1 arrest [27–29]. Additionally, that overexpression of p21 arrests the cell-cycle transition from the G1- to the S-phase via inhibition of CDK2 activity, expression of p21 was greater, and simultaneously the expression of CDK2 was lower which has also been documented [30]. Further, it has been found that, in inhibited cell proliferation, the levels of p21 increased and the levels of Cyclin E and CDK2 decreased [31]. Others have found that virus-induced cell cycle arrest induced p53 and p21 accumulation and decreased phosphorylation of Rb [26]. Those studies suggest that the p53/p21 pathway, by upregulated p53, p21 and downregulated cyclins, CDK, and p-Rb, plays an important role in regulating the cell senescent process. Some researchs have been reported that the protein and its function were based on a subcellular localization [29]. The main role of p53 and p21 proteins that are accumulated in the nucleus is the cell cycle arrest response to genotoxic stress such as DNA damage. In the nucleus, p53 works as a transcriptional factor and regulates transactivation of several proteins, including the p21 [32]. It has been reported that the presence of p21 protein in the nucleus is necessary for cell cycle arrest. In the nucleus, p21 binds to and inhibits the activity of cyclin-dependent kinases and blocks the transition from G1 phase into S-phase [29]. Others have found that nuclear CDK2 is associated with proliferation [33]. Because of the prominent role that p53 has in the DNA damage response of differentiated cells, it is most likely that p53 has a similar function in stem cells. It has also been reported that after DNA damage, nuclear accumulation and activation of p53 were found in stem cells. Solozobova et al. have found that, in proliferating embryonic stem cells, p53 is localized predominantly in the cytoplasm. DNA damage-induced nuclear accumulation of p53 in embryonic stem cells activates transcription of the target gene p21 [32, 34]. These results suggested that p53, p21, and CDK2 exert function were based on their subcellular localization.

Numerous studies have shown that the p53/p21 is pathway involved in regulating MSCs senescence [14, 15, 17, 35, 36]. It has been found that p53 regulates the proliferation of MSCs [16]. The expressions of both p53 and p21 were significantly upregulated with diminished capacities for proliferation in old rhesus monkeys BM-MSCs compared to those from young donors [37]. Furthermore, the late-passage MSCs showed increased expression of p21 and decreased proliferation capacity has also been documented [38]. A line of evidence indicated accelerating MSCs proliferation by downregulation of p21 [38, 39]. Emerging evidence indicates that, transforming growth factor *β*-induced BM-MSCs senescence through the increased of expressions of p53 and p21, low expression of p-Rb, after treatment with cell growth factors, the cell growth arrest was suppressed through the suppression of p21 and p53 expression levels and the increase of p-Rb expression levels [40]. In addition, MSCs treated with small interfering RNA targeting p21 were demonstrated proliferation significantly faster than control cells [39], while it has been documented that knockdown p21 enhances proliferation, the expression of stemness markers, and osteogenic potential in human MSCs [38]. It has also been demonstrated that p21−/− mice had significantly less radiation damage, including 40% increased growth potential. They also have found that p21−/− MSCs had 4-fold greater proliferation rate and nearly 7-fold lower senescence as compared to control MSCs [41]. These results suggested that the p53/p21 pathway plays an important role in regulating BM-MSCs cell senescence process. In the BM-MSCs from SLE patients, we observed that the expressions of p53 and p21 were increased, while the levels of Cyclin E and CDK2 were strongly decreased, and the phosphorylation of Rb was also decreased (Figure 2). To further confirm the role of the p53/p21 pathway in regulating senescence process of BM-MSCs from SLE patients, we used MSCs transfected with p21 siRNA inhibition of p21 expression in BM-MSCs from SLE patients. We found that the morphology of BM-MSCs from SLE patients showed more spindle-shaped fibroblast-like growth, increased proliferation rate, increased CFU, less SA-*β*-gal-positive cells, and more cells restricted in the S-phase harvested from SLE patients' BM-MSCs after knockdown of p21 expression. We found that Cyclin E and CDK2 were increased in SLE patients' BM-MSCs culture with siRNA p21, and p-Rb also increased in the knockdown p21 of BM-MSCs (Figure 6). Interestingly, we also found that more expressions and more location of p53 and p21 were found in nuclear whereas lower levels were found in the cytoplasm of the BM-MSCs from the SLE patients than that of the normal control (Figure 3). Furthermore, Cyclin E and CDK2 were mainly localized and more expressed in the cytoplasm while lower levels were found in the nuclei of the BM-MSCs from the SLE patients than those of the normal control (Figure 4). These data indicated that the p53/p21 pathway plays an essential role in the ageing process of BM-MSCs from SLE patients.

In conclusion, in the present study, we have further determined that the BM-MSCs from the SLE patients were senescent MSCs; the abilities of proliferation and colony form were suppressed. The p53/p21 pathway plays a key role in the regulation of cell senescence process of BM-MSCs from SLE patients. These findings could explore the mechanism of cell senescence of BM-MSCs in SLE patients.

## Figures and Tables

**Table 1:** Clinical features of 22 SLE Patients.

Patients	Age (years) and sex	Disease duration	Current treated	SLEDAI
1	19 F	2 y	Pred: 20–40 mg/day; CTX: 0.4 g/2 weeks HCQ: 0.2/day; MMF: 1.5–2.0 g/day	30
2	22 F	1.5 y	Pred: 15–20 mg/day CTX: 0.4 g/2 weeks; HCQ: 0.2/day	24
3	39 F	10 y	Pred: 10 mg/day; HCQ: 0.2/day	8
4	37 F	8 y	Pred: 15–20 mg/day LEF: 0.2 g/day; HCQ: 0.2/day	22
5	24 F	1 y	Pred: 15 mg/day	8
6	42 F	6 y	Pred: 20–30 mg/day HCQ: 0.4/day; CTX: 0.6 g/3 weeks	26
7	28 F	1 y	Pred: 10 mg/day; HCQ: 0.2/day	12
8	23 F	1 y	Pred: 5–7.5 mg/day HCQ: 0.2/day; CTX: 0.4 g/4 weeks	18
9	32 F	3 y	Pred: 5–10 mg/day LEF: 0.2 g/day; HCQ: 0.2/day	12
10	25 F	4 y	Pred: 5–7.5 mg/day LEF: 0.2 g/day; HCQ: 0.2/day	16
11	14 F	2 y	Pred: 5–10 mg/day HCQ: 0.2/day; MMF: 1.5–2.0 g/day	9
12	21 F	2 m	Pred: 7.5–10 mg/day MMF: 1.5–2.0 g/day; HCQ: 0.2/day	19
13	32 F	4 m	Pred: 5–7.5 mg/day LEF: 0.2 g/day; HCQ: 0.2/day	16
14	20 F	7 m	Pred: 10–15 mg/day LEF: 0.2 g/day; HCQ: 0.2/day	21
15	24 F	2 m	Pred: 7.5–10 mg/day LEF: 0.2 g/day; HCQ: 0.2/day	18
16	22 F	3 m	Pred: 30–40 mg/day MMF: 1.5–2.0 g/day; HCQ: 0.4/day	26
17	15 F	1 m	Pred: 5 mg/day; HCQ: 0.2/day	8
18	33 F	4 m	Pred: 5–7.5 mg/day LEF: 0.2 g/day; HCQ: 0.2/day	12
19	36 F	3 m	Pred: 7.5 mg/day; HCQ: 0.2/day	9
20	23 F	8 m	Pred: 20–40 mg/day CTX: 0.4 g/2 weeks; HCQ: 0.2/day	17
21	20 F	4 m	Pred: 20–30 mg/day CTX: 0.4 g/4 weeks; HCQ: 0.2/day	14
22	29 F	6 m	Pred: 20–40 mg/day CTX: 0.4 g/2 weeks; HCQ: 0.2/day	20

y: years; m: mouth; S: skin; J: joints; H: hematologic; M: myositis; V: vasculitis; R: renal; Pred: prednisolone; HCQ: hydroxychloroquine; CTX: cyclophosphamide; LEF: Leflunomide; MMF: Mycophenolate Mofetil.
